# Whole-Genome Sequence of a Novel Hantavirus Isolated from the European Mole (*Talpa europaea*)

**DOI:** 10.1128/genomeA.00508-15

**Published:** 2015-05-28

**Authors:** Se Hun Gu, Janusz Hejduk, Janusz Markowski, Marcin Markowski, Paweł P. Liberski, Richard Yanagihara

**Affiliations:** a Pacific Center for Emerging Infectious Diseases Research, John A. Burns School of Medicine, University of Hawaii at Manoa, Honolulu, Hawaii, USA; b Department of Teacher Training and Biodiversity Studies, Faculty of Biology and Environmental Protection, University of Łódź, Łódź, Poland; c Department of Experimental Zoology and Evolutionary Biology, Faculty of Biology and Environmental Protection, University of Łódź, Łódź, Poland; d Department of Molecular Pathology and Neuropathology, Medical University of Łódź, Łódź, Poland

## Abstract

The complete genome sequence of Nova virus, a novel hantavirus isolated from a European mole (*Talpa europaea*) captured in central Poland, was determined. The availability of this sequence will facilitate the search for other mole-borne hantaviruses and will accelerate the acquisition of new knowledge about their phylogeography and evolutionary origin.

## GENOME ANNOUNCEMENT

Among the five genetically distinct hantaviruses (family *Bunyaviridae*, genus *Hantavirus*) discovered hitherto in moles (order *Eulipotyphla*, family *Talpidae*) from Europe, Asia, and North America (1), only one, Rockport virus, which is harbored by the eastern mole (*Scalopus aquaticus*) in the United States (2), has been fully sequenced. A sequence alignment and comparison of Rockport virus and the partial genomes of other mole-borne hantaviruses indicate that Nova virus (NVAV), originally detected in archival liver tissue of a European mole (*Talpa europaea*) captured in Zala County, Hungary, in 1999 (3) and subsequently shown to be widespread in France (4) and Poland (5), appears to be the most divergent (3–5). However, the unavailability of the full-genome sequence of NVAV has impeded progress in ascertaining the evolutionary history of hantaviruses.

Here, we report the full-length genome sequence of NVAV strain Te34 (also known as strain 3483), isolated in Vero E6 cells (CRL 1586; American Type Culture Collection, Manassas, VA) from lung tissue of an adult male European mole, captured in Huta Dłutowska (51°35′49.51″N 19°22′46.80″E) near Łódź in central Poland on 26 August 2013. Total RNA, which was extracted from infected Vero E6 cells and original lung tissue using the PureLink Micro-to-Midi total RNA purification system (Invitrogen, San Diego, CA), was reverse transcribed using the SuperScript III first-strand synthesis systems (Invitrogen) with random hexamers. The negative-sense single-stranded tripartite RNA genome of NVAV was amplified using oligonucleotide primers designed with the MegAlign Clustal W program (DNAStar, Inc., Madison, WI). The 5′ and 3′ termini of each segment were amplified using the 3′-Full RACE core set (TaKaRa Bio, Inc., Otsu, Japan). For taxonomic verification of the mole host, the 1,140-nucleotide cytochrome *b* gene of mitochondrial DNA was amplified by PCR and sequenced (3).

The full-length L-genomic segment of NVAV strain Te34 was 6,563 nucleotides, with a predicted RNA-dependent RNA polymerase (RdRp) of 2,157 amino acids, starting at nucleotide position 34 and including 56 nucleotides of the 3′ noncoding region. The entire M-genomic segment of NVAV Te34 was 3,590 nucleotides, with a predicted glycoprotein precursor (GnGc) of 1,127 amino acids. The highly conserved WAASA amino acid motif of the M segment was found at amino acid positions 641 to 645. The full-length 1,825-nucleotide S-segment sequence of the NVAV isolate and the NVAV in the original lung tissue were identical, containing a single open reading frame with a predicted nucleocapsid (N) protein of 428 amino acids (nucleotide positions 53 to 1339), and 52- and 486-nucleotide 5′ and 3′ noncoding regions, respectively. A comparison of the S segments between the NVAV isolate and the prototype NVAV strain MSB95703 from Hungary showed nucleotide and amino acid sequence similarities of 85.6% and 96.5%, respectively. The secondary structure of the predicted N protein of NVAV strain Te34 was virtually identical to that reported earlier for the prototype NVAV strain (3).

### Nucleotide sequence accession numbers. 

The GenBank accession numbers for the genome sequences of the NVAV Te34 isolate are KR072621, KR072622, and KR072623 for the S, M, and L segments, respectively. The GenBank accession number for the corresponding S segment from the original lung tissue is KM403445.
